# Tissue- and Cell-Specific Cytokinin Activity in *Populus* × *canescens* Monitored by *ARR5::GUS* Reporter Lines in Summer and Winter

**DOI:** 10.3389/fpls.2016.00652

**Published:** 2016-05-13

**Authors:** Shanty Paul, Henning Wildhagen, Dennis Janz, Thomas Teichmann, Robert Hänsch, Andrea Polle

**Affiliations:** ^1^Department of Forest Botany and Tree Physiology, Georg-August-Universität GöttingenGöttingen, Germany; ^2^Department of Molecular and Cell Biology of Plants, Institute for Plant Biology, University of TechnologyBraunschweig, Germany

**Keywords:** cytokinin, localization, *ARR5*, summer, winter, dormancy, wood

## Abstract

Cytokinins play an important role in vascular development. But knowledge on the cellular localization of this growth hormone in the stem and other organs of woody plants is lacking. The main focus of this study was to investigate the occurrence and cellular localization of active cytokinins in leaves, roots, and along the stem of *Populus* × *canescens* and to find out how the pattern is changed between summer and winter. An *ARR5::GUS* reporter construct was used to monitor distribution of active cytokinins in different tissues of transgenic poplar lines. Three transgenic lines tested under outdoor conditions showed no influence of *ARR5::GUS* reporter construct on the growth performance compared with the wild-type, but one line lost the reporter activity. *ARR5::GUS* activity indicated changes in the tissue- and cell type-specific pattern of cytokinin activity during dormancy compared with the growth phase. *ARR5::GUS* activity, which was present in the root tips in the growing season, disappeared in winter. In the stem apex ground tissue, *ARR5::GUS* activity was higher in winter than in summer. Immature leaves from tissue-culture grown plants showed inducible *ARR5::GUS* activity. Leaf primordia in summer showed *ARR5::GUS* activity, but not the expanded leaves of outdoor plants or leaf primordia in winter. In stem cross sections, the most prominent *ARR5::GUS* activity was detected in the cortex region and in the rays of bark in summer and in winter. In the cambial zone the *ARR5::GUS* activity was more pronounced in the dormant than in growth phase. The pith and the ray cells adjacent to the vessels also displayed *ARR5::GUS* activity. *In silico* analyses of the tissue-specific expression patterns of the whole PtRR type-A family of poplar showed that *PtRR10*, the closest ortholog to the *Arabidopsis ARR5* gene, was usually the most highly expressed gene in all tissues. In conclusion, gene expression and tissue-localization indicate high activity of cytokinins not only in summer, but also in winter. The presence of the signal in meristematic tissues supports their role in meristem maintenance. The reporter lines will be useful to study the involvement of cytokinins in acclimation of poplar growth to stress.

## Introduction

Cytokinins are adenine derivatives that act as master regulators of plant growth and development. They are synthesized mainly in the root tips (Dieleman et al., 1997; Miyawaki et al., 2004; Aloni et al., 2005), but also locally in shoot tissues (Sakakibara, 2006; Tanaka et al., 2006; Hirose et al., 2008; Kamada-Nobusada and Sakakibara, 2009). Root-derived cytokinins are transported acropetally through xylem sap by the transpirational pull (Aloni et al., 2005), while shoot-derived cytokinins are transported through phloem (Bishopp et al., 2011). Active and inactive forms of cytokinins occur as free bases and as ribosides, ribotides, or glucose conjugates, respectively (Mok and Mok, 2001; Romanov et al., 2006).

Cytokinins have roles in almost all aspects of plant growth and development including cell division, shoot initiation and growth, sink/source relationships, nutrient uptake, breaking of bud dormancy, delay of leaf senescence, and regulation of vascular development (Hwang et al., 2012; Kieber and Schaller, 2014). Cytokinins determine vascular cell identities, except those of the protoxylem (Mähönen et al., 2000; Hutchison et al., 2006; Yokoyama et al., 2007; Argyros et al., 2008) and promote the development of vascular cambium (Matsumoto-Kitano et al., 2008; Nieminen et al., 2008). Cytokinins specify the vascular pattern by regulating the level of PIN auxin eﬄux proteins (Bishopp et al., 2011). Cytokinins increase the sensitivity of the cambium to the auxin signal thereby determining wood quantity and quality (Aloni, 1991, 2001).

Cytokinin perception and signaling in plants has been extensively studied in *Arabidopsis* and involves a His-Asp phosphorelay that mediates the signal transmission (Mizuno, 2005; Schaller et al., 2008). Among the response regulators in this pathway, type-A *ARRs* (*Arabidopsis* Response Regulators), i.e., genes which contain the highly conserved Lys and two Asp residues in their receiver domains, are the primary response genes for cytokinins (D’Agostino et al., 2000). Ten type-A *ARR* genes are described in *Arabidopsis* (D’Agostino et al., 2000; Schaller et al., 2008; Pils and Heyl, 2009) and eleven in *Populus trichocarpa* (Ramírez-Carvajal et al., 2008; Immanen et al., 2013). The *ARR* genes are transcriptionally regulated and can be induced by exogenous cytokinin treatment (D’Agostino et al., 2000; Taniguchi et al., 1998).

In trees, changes in endogenous cytokinin levels in relation to seasonality have been studied for a long time. Most of these studies focused on the endogenous cytokinin levels in xylem or phloem sap of the trees (Hewett and Wareing, 1973; Alvim et al., 1976; Weiler and Ziegler, 1981; Tromp and Ovaa, 1990; Cook et al., 2001) or reported the endogenous cytokinin concentrations in different organs (Hewett and Wareing, 1973; Van Staden and Dimalla, 1981; Cook et al., 2001). Furthermore active and inactive forms of cytokinins were distinguished (Hewett and Wareing, 1973; Van Staden and Dimalla, 1981; Tromp and Ovaa, 1990) and their changes were related to seasonal fluctuations (Tromp and Ovaa, 1990). For example, in the xylem sap of apple trees, the active *trans*-zeatin type (tZ) levels were high during the growing season, dropped during dormancy and showed an increase during bud burst, whereas continued to increase during the growing season (Tromp and Ovaa, 1990). Despite the importance of cytokinins in vascular development, knowledge on the cellular localization of this growth hormone in the stem and other organs of woody plants is still lacking. Furthermore, it is unclear how the tissue-specific distribution of active cytokinins is influenced by dormancy.

The goal of this study was to investigate the occurrence and cellular localization of active cytokinins in leaves, roots and along the stem of poplar and to find out how the pattern is changed between the active growth phase in summer and dormancy in winter. Our hypothesis was that cytokinin activity was present in actively growing tissues in summer and lacking in winter, except in those tissues, where cells have to be kept in the meristematic stage. Tissue-specific localization patterns of cytokinin activity were also compared with expression of genes belonging to the type-A Response Regulator (RR) family in poplar. In *Arabidopsis*, the *ARR5::GUS* (β-glucuronidase) reporter construct has been used to monitor the distribution of active cytokinins in different tissues (D’Agostino et al., 2000). *ARR5* has high homology to the cytokinin-inducible gene *PtRR10* of *Populus trichocarpa* (Ramírez-Carvajal et al., 2008; Immanen et al., 2013). Here, we employed the *ARR5::GUS* construct as a tool to investigate the localization pattern of active cytokinins in poplar. The transgenic poplar cytokinin reporter lines were grown outdoors under ambient conditions and used to map *ARR5* activity in summer and winter.

## Materials and Methods

### Plant Transformation

The *ARR5::GUS* construct described in D’Agostino et al. (2000) was provided by Prof. Kieber (University of North Carolina, Chapel Hill, NC, USA), cloned, transformed into *Agrobacterium tumefaciens* strain C58C1/MP90 and then used to transform *Populus* × *canescens* [INRA (Institut National de la Recherche Agronomique) clone 717-1B4] as described by Teichmann et al. (2008). Plantlets were regenerated, maintained on Murashige and Skoog (MS) medium containing 50 mg l^-1^ kanamycin and micropropagated after Leple et al. (1992).

### Selection of Transgenic Reporter Lines

**FIGURE 1:**
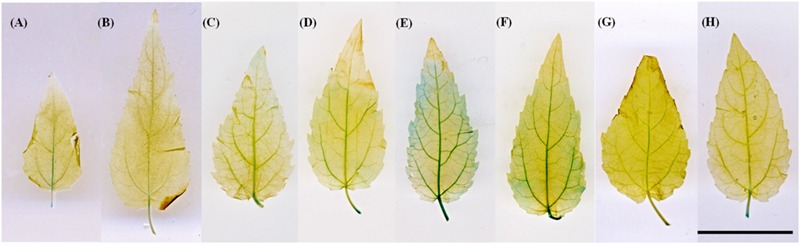
***ARR5::GUS* activity in leaves of *ARR5::GUS* poplar reporter line.** The leaves of line 80 were incubated directly in GUS staining solution **(A,B)** or GUS staining was performed after mock-incubated in 0.1% DMSO (solvent control; **C,D**). **(E,F)** show *ARR5::GUS* activity in leaves after petiole-feeding with 5 μM thidiazuron and BAP, respectively, while **(G,H)** show *ARR5::GUS* activity in leaves after petiole feeding with 5 μM adenine in the light (200 μmol quanta m^-2^ s^-1^ PAR) for 24 h. Scale bar = 2 cm

Leaves from 3-week-old transformed plantlets were collected and GUS staining was performed according to Jefferson et al. (1987) as modified by Teichmann et al. (2008). Briefly, the presence of GUS activity was investigated in intact leaves that were vacuum-infiltrated with GUS buffer (100 mM NaH_2_PO_4_, pH 7.0, 10 mM Na_4_EDTA, 0.05% Triton X-100) containing 1 mg ml^-1^ 5-bromo-4-chloro-3-indolyl-β-D-glucuronic acid (Duchefa, Haarlem, The Netherlands). The leaves were then incubated in the dark at 37 °C for 24 h and chlorophyll was removed by ethanol treatment. The *ARR5::GUS* activity was observed mainly in the petiole and primary veins of these leaves. The leaves were viewed and photographed directly. From the regenerated plantlets which were maintained on MS medium containing kanamycin, 17 lines showed GUS activity after GUS staining, mainly in the veins (**Figures 1A,B**). The pattern was similar to that of mock treated leaves (**Figures 1C,D**).

All plantlets from these 17 lines showed similar morphology and growth *in vitro* when compared to the wild-type (WT). Plantlets from each line were propagated *in vitro*. The leaves from these lines were also treated with an exogenous cytokinin supply in order to monitor the response of *ARR5::GUS* construct to cytokinin. For this purpose, leaves from 3-week-old *in vitro* micropropagated plantlets were fed by the petioles with a solution containing 5 μM thidiazuron (Duchefa, Haarlem, The Netherlands) or 5 μM 6-benzylaminopurine (BAP, Sigma–Aldrich Chemie, Steinheim, Germany) in 0.1% DMSO (Merck KGaA, Darmstadt, Germany), both active cytokinin analogs or 5 μM adenine (Sigma–Aldrich Chemie, Steinheim, Germany), which is an inactive cytokinin analog. A solution of 0.1 % DMSO (Merck KGaA, Darmstadt, Germany) was used as the solvent control. During the treatment the leaves were kept inside a sealed glass jar at high humidity to avoid desiccation stress. The leaves were allowed to transpire under 200 μmol quanta m^-2^ s^-1^ photosynthetically active radiation (PAR) for 24 h. Afterward, the leaves were directly used for GUS staining as above. Examples for cytokinin induction of the *ARR5* promoter in thidiazuron- or BAP-fed leaves are shown in **Figures 1E,F**. When compared to controls, the thidiazuron-treated leaves showed a strong induction in *ARR5::GUS* activity over the leaf blade and in tertiary veins (**Figure 1E**). On the other hand, the leaves treated with the inactive cytokinin analog adenine did not show an induction (**Figures 1G,H**). For documentation of staining pattern, the leaves were laid flat in a petri dish filled with distilled water and were scanned (Canoscan 4400F, Canon Inc., China).

From the lines that showed an increased *ARR5::GUS* activity under exogenous cytokinin treatment, three lines (line 9, 32, and 80) were selected for the study.

### Plant Cultivation

*In vitro* micropropagated plantlets of the lines 9, 32, and 80 along with WT, were grown in hydroponics for 3 weeks and then transferred into pots with soil (Fruhstorfer Erde Type N, Hawite Gruppe GmbH, Vechta, Germany) as described by Müller et al. (2013). The plants were grown in a greenhouse for 3 months under controlled environmental conditions: 16 h day length, 200 μmol quanta m^-2^ s^-1^ PAR, 20 °C air temperature and 55% relative air humidity. Afterward, the potted plants were transferred from the greenhouse to a caged area outdoors (Göttingen, Germany, 51.55739°N, 9.95857°E, 293 m above sea level) and acclimated to ambient light and temperature (after Müller et al., 2013). On 18th July 2011, the poplars were planted in four boxes (3.5 m length × 3 m width × 0.7 m height) filled with a compost soil and sand mixture (1:1) (Vogteier Erdenwerk GmbH, Niederdorla, Germany). The WT and transgenic lines were planted in a mixed design. Each box was equipped with a total of 42 plants comprising 10 plants each of WT, line 9 and 80, and 12 plants of line 32. The first row of plants near to the box edges was not included in any of the analyses to avoid edge effects.

During the growing season, plants were watered with tap water every second day or daily on warm days. Air temperature, relative humidity, and PAR for every hour per day were recorded during the whole study period using MeteoLOG TDL 14 data logger (Adolf Thies GmbH & Co. KG, Göttingen, Germany).

### Harvest

Harvests were conducted in the growing season (August, 2012) when the mean temperature was 22.4 °C and during dormancy (January, 2013) when the mean temperature was -5.0 °C. In the growing season harvest, four plants each from WT, line 9, 32, and 80 were harvested. In the dormancy harvest, one plant from WT and two plants from each line 9, 32, and 80 were harvested. Roots, bark, wood, and leaves were separated and fresh mass was determined for each fraction. Aliquots of these plant tissues were oven dried at 60 °C for 7 days for measurement of dry mass. Tissue dry mass (g) was calculated as:

$$\frac{Dry mass of the aliquot(g)\;\times total tissue fresh mass(g)}{fresh mass of the aliquot(g)}$$

During harvest, the following fresh tissues were collected for GUS staining: one half of the apical bud, leaf disks (diameter 5 mm) from the first fully developed leaf from the apex, stem cross sections (2 mm thickness) at three positions: top (50 mm beneath the stem apex), middle (the position in the stem exactly in the center between the apex and the shoot–root junction) and bottom (50 mm above the root–shoot junction), and fine root tips. The stem cross sections were cut using a micro-saw (Proxxon, Föhren, Germany). The materials were directly transferred into the GUS buffer and GUS staining was performed as described above.

### GUS Activity Analyses at Tissue and Cellular Level

Tissue staining patterns were documented by photos, which were taken with a digital camera (DFC420 C, Leica Microsystems Ltd., Germany) attached to a stereomicroscope (M205 FA, Leica Microsystems Ltd., Wetzlar, Germany). The stained tissues were fixed in a solution of 1 part of 37% formaldehyde, 1 part of 100% acetic acid, and 18 parts of 70% ethanol. Subsequently, the fixed tissues were dehydrated in a series of ethanol solutions (70, 80, 90, and 96% (v/v)) for 2 h each at room temperature. The tissues were embedded in Technovit 7100 resin (Heraeus Kulzer GmbH & Co. KG, Germany) according to manufacturer’s instructions with the following modifications: The dehydrated samples were infiltrated in 1:1 (v/v) solutions of 96% ethanol and Technovit 7100 basic solution for 5 h. Then the samples were infiltrated in 1:2 and then in 1:3 (v/v) solutions of 96% ethanol and Technovit 7100 basic solution for 12 and 5 h, respectively. Thereafter, the samples were treated with Technovit 7100 infiltration medium consisting of 1 g Hardner I in 100 ml Technovit 7100 basic solution (provided by the manufacturer) for 24 h. A reduced pressure of 20 kPa for 15 min was applied at each step during infiltration. Finally, the tissues were embedded in the embedding medium (prepared by mixing 30 ml infiltration medium and 1.5 ml Hardner II provided by the manufacturer). Sections of 15 μm thickness were cut with a rotarymicrotome (RM 2265, Leica Microsystems, Wetzlar, Germany) and viewed under a microscope (Axioplan Observer.Z1, Carl Zeiss GmbH, Germany). Photographs were taken at 100× and 200× magnification with a digital camera (Axio Cam MRC, Carl Zeiss Microimaging GmbH, Göttingen, Germany) attached to the microscope (Axioplan Observer.Z1, Carl Zeiss GmbH, Germany).

### Gene Expression Analyses of PtRR Type-A Family

For the analysis of tissue specific expression patterns of poplar genes belonging to the two component RR type-A gene family of cytokinin signaling pathway, the gene list as reported by Ramírez-Carvajal et al. (2008) was used. The genomic sequence of each gene was obtained from Joint Genome Institute, JGI^1^ and the respective gene IDs were obtained by blasting the genomic sequences in Phytozome v10.1^2^ (Goodstein et al., 2012). The homolog of each gene in *Arabidopsis* was obtained by blasting the protein sequence taken from Phytozome, in TAIR^3^. The gene names and gene IDs of the genes used for the expression analyses have been compiled in **Table 1**. Microarrays were downloaded from the EMBL-EBI ArrayExpress database (Kolesnikov et al., 2015). For *P.* × *canescens*: E-GEOD-16495 (shoot apex; Yordanov et al., 2014), E-MEXP-1928 (mature leaves; Janz et al., 2010), E-MEXP-2120 (mature leaves), E-MEXP-3741(bark; He et al., 2013), E-MEXP-2031 (developing xylem; Janz et al., 2012), E-GEOD-33977 (rays- summer and winter; Larisch et al., 2012), E-MEXP-1874 (fine roots; Luo et al., 2009), E-GEOD-43162 (fine roots; Wei et al., 2013), and for *P. trichocarpa*: E-GEOD-30507 (stem, shoot and leaf primordia, mature leaves, developing xylem, cambium, bark; Ko et al., 2012), E-MEXP-3910 (young leaves; Bai et al., 2013), E-GEOD-49983 (bark), E-MTAB-1483 (developing xylem and elongation zone; Euring et al., 2014), E-GEOD-21480 (stem- summer and winter); E-MEXP-3909 (young roots; Bai et al., 2013).

**Table 1:** Poplar genes belonging to the two component type-A response regulator gene family (Ramírez-Carvajal et al., 2008) that were used for analysis of tissue-specific expression pattern.

*Populus trichocarpa* gene name	*Populus trichocarpa* gene ID	*Arabidopsis* gene name	AGI
*PtRR1*	Potri.010G037800	*ARR3*	AT1G59940
*PtRR2*	Potri.008G193000	*ARR3*	AT1G59940
*PtRR3*	Potri.002G082200	*ARR9*	AT3G57040
*PtRR4*	Potri.003G197500	*ARR9/ARR8*	AT3G57040/ AT2G41310
*PtRR5*	Potri.001G027000	*ARR9/ARR8*	AT3G57040/ AT2G41310
*PtRR6*	Potri.006G041100	*ARR9*	AT3G57040
*PtRR7*	Potri.016G038000	*ARR8*	AT2G41310
*PtRR8*	Potri.019G058900	*ARR17*	AT3G56380
*PtRR9*	Potri.013G157700	*UCP030365*	AT5G05240
*PtRR10*	Potri.015G070000	*ARR5*	AT3G48100
*PtRR11*	Potri.019G133600	*ARR17*	AT3G56380

For the annotation of the microarray ID to the best gene model, the annotation file downloaded from Aspen Database (Tsai et al., 2011) was used.

### Statistical Analyses

Statistical analyses were performed using the free statistical software R (version 3.1.1, R Core Team, 2014). One-way ANOVA was conducted for dry biomass data with plant lines (transgenic reporter lines and WT) as factor. Normality and homogeneity of variance were tested visually by plotting residuals and the data was transformed logarithmically (log_2_) if needed. Data shown are mean ± SE. Means were considered to be significantly different with a *p*-value ≤ 0.05.

For the analyses of expression data, to summarize and normalize the array probes, ‘rma’ function from the R package ‘affy’ (Gautier et al., 2004) obtained from Bioconductor (Kauffmann et al., 2009) was used. Mean transcript abundance of the biological replicates was calculated for each gene in each tissue. When more than one probe set was present for one gene, all probe sets were used to calculate the mean value. The means were used for creating a heatmap with the ‘heatmap.2’ function from the R package ‘gplots’ (Warnes et al., 2012).

## Results

### The *ARR5::GUS* Reporter Lines Showed No Growth Differences Compared to Wild-type Poplars

**FIGURE 2:**
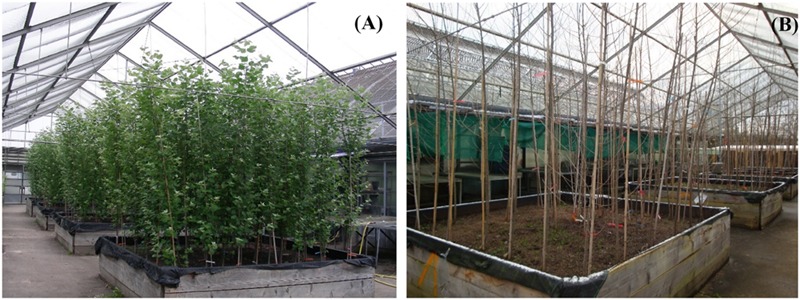
***Populus × canescens* wild-type (WT) and *ARR5::GUS* transgenic reporter lines before harvest in the growth phase (**A**; August, 2012) and before harvest during dormancy (**B**; January, 2013)**.

The poplar lines 9, 32, and 80 were grown in ambient conditions, along with WT plants for 1.5 years. The determination of the dry mass did not show any significant difference among the lines (**Table 2**). There were no apparent visual differences neither in summer nor in winter (**Figure 2**) suggesting that the transformation with the *ARR5::GUS* gene construct did not hit any gene that was relevant for growth and that the expression of the construct had no influence on the plant stature.

**Table 2:** Biomass of 1-year-old *Populus* × *canescens* wild-type and *ARR5::GUS* reporter lines in the growing season.

	WT	Line 9	Line 32	Line 80
**Parameter**				
Stem + branches (g dry wt)	162.1 ± 18.1	124.0 ± 16.2	159.2 ± 38.2	228.0 ± 13.8
Coarse root (g dry wt)	74.8 ± 12.3	59.8 ± 10.6	63.7 ± 10.1	84.7 ± 3.3
Fine root (g dry wt)	5.7 ± 1.0	4.7 ± 0.6	6.6 ± 1.3	5.3 ± 1.0
Below-ground (g dry wt)	80.4 ± 12.4	64.5 ± 10.8	70.3 ± 11.2	90.0 ± 2.3

*One-year-old whole poplar trees were harvested in August, 2012. Data indicate mean ± SE (*n* = 4). One-way ANOVA conducted for dry mass of the plant lines did not reveal any significant differences (*p* > 0.05).*

### *ARR5::GUS* Activity Reports a Tissue- and Cell-type Specific Pattern of Cytokinin Activity in Poplar in the Growth Phase

The localization pattern of *ARR5::GUS* activity during growing season was investigated in apical buds, leaf disks, root tips and in stem cross sections at three positions, i.e., top, middle, and bottom. Plants from line 9 did not display a *GUS* signal in any of the samples from the outdoor grown plants, suggesting that silencing had occurred. Therefore, the pictures of these samples were not considered.

**FIGURE 3:**
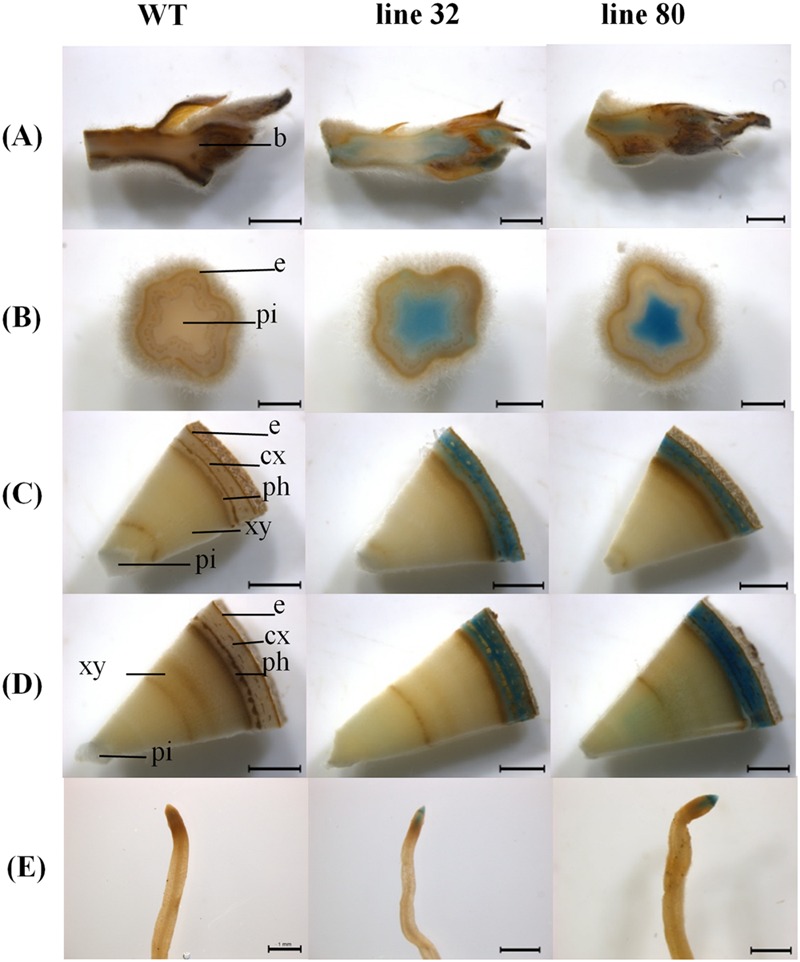
***ARR5::GUS* activity observed in different tissues of *Populus × canescens* reporter lines and WT in the growing season.** Representative pictures of *n* = 4 replicates per line are shown. Rows **(A–E)** represent apical bud, stem sections from top, middle, and bottom positions, and the root tips, respectively. Tissues are indicated by the following abbreviations: b, apical bud base; e, epidermis; cx, cortex; ph, phloem; xy, xylem; and pi, pith. Scale bar = 2 mm for apical bud, stem middle and stem bottom. Scale bar = 1 mm for stem top and root tip.

**FIGURE 4:**
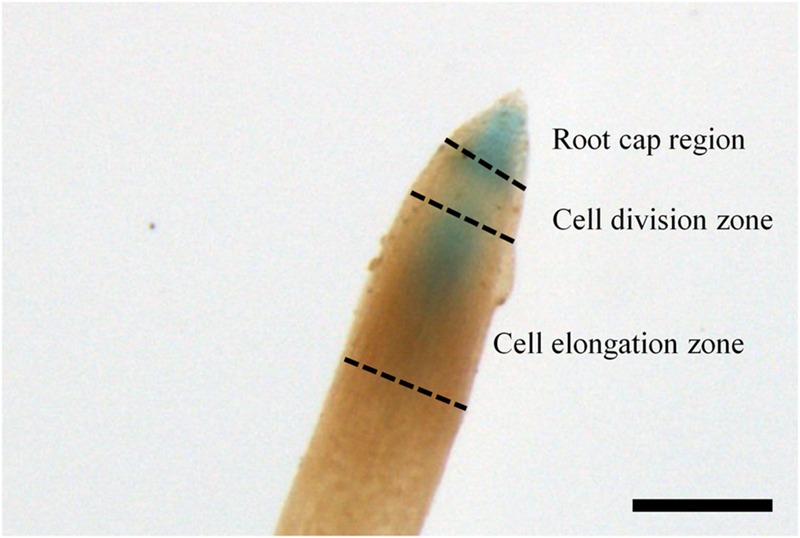
***ARR5::GUS* activity in the root tip of the *Populus × canescens* reporter line 32 during the growth phase.** Scale bar = 250 μm.

In the growing season, line 32 and 80 exhibited *ARR5::GUS* activity in all tissues (**Figure 3**), except in mature leaves (not shown). In the apical buds, the *ARR5::GUS* activity was localized in the leaf primordia and also in the apical bud base from where the leaf primordia started (**Figure 3A**). *ARR5::GUS* was also expressed in root tips (**Figure 3E**). Higher magnification showed that the staining was concentrated in the root cap region, decreased in the cell division zone and was stronger again at the onset of the cell elongation zone (**Figure 4**). The signal showed a gradual decrease toward the direction of the shoot (**Figure 4**).

Examination of cytokinin activity along the stem revealed strong staining in pith in the region of the stem elongation zone, while the signal in the pith disappeared in the stem middle and at the stem bottom, where the pith was compressed by secondary growth (**Figures 3B–D**). In the stem middle and at the bottom the bark region below the periderm also showed a strong *GUS* staining (**Figures 3C,D**).

**FIGURE 5:**
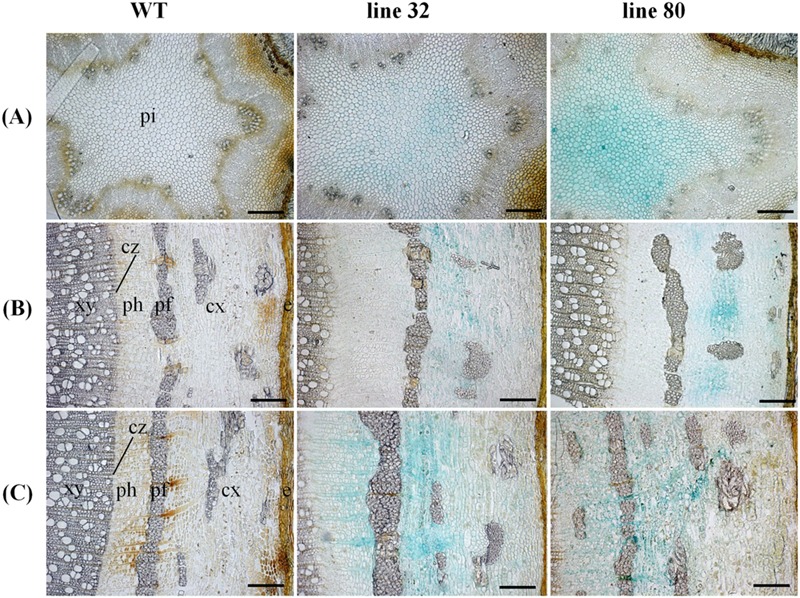
**Cellular localization of *ARR5::GUS* activity in different stem sections of *Populus × canescens* reporter lines and the WT in the growth phase.** Representative pictures of *n* = 4 replicates per line are shown. Rows **(A–C)** represent stem top, stem middle, and stem bottom sections, respectively. Here, e, epidermis; cx, cortex; pf, phloem fibers; ph, phloem; cz, cambial zone; xy, xylem; and pi, pith. Scale bar = 200 μm.

**FIGURE 6:**
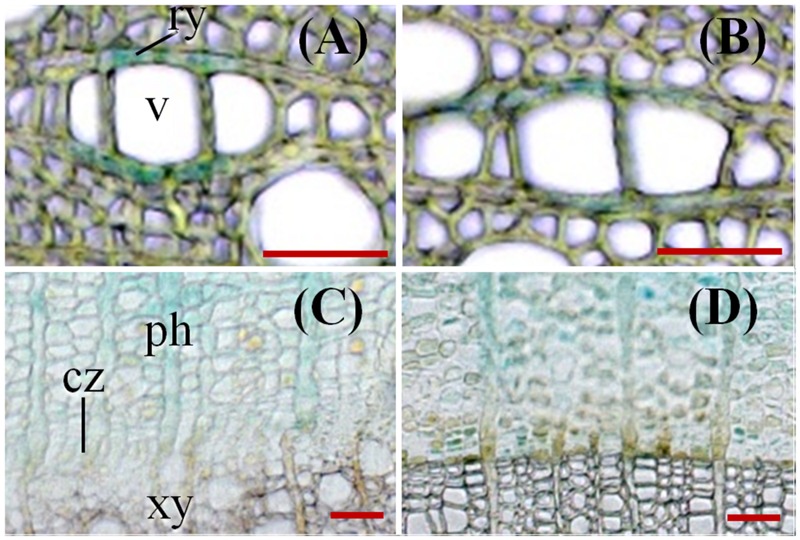
***ARR5::GUS* activity observed in the xylem rays **(A,B)** and in cambium **(C,D)** at the stem bottom of *Populus × canescens* in the growth phase and during dormancy. (A,C)** represent samples from growth phase and **(B,D)** represent samples during dormancy of line 32. The following abbreviations were used: ry, ray cell; v, vessel; ph, phloem; cz, cambial zone; and xy, xylem. Scale bar = 50 μm.

To investigate the cellular localization pattern of *ARR5::GUS* activity, stem cross sections were analyzed at a higher magnification. In the elongation zone strong *ARR5::GUS* activity in the pith was confirmed, but no staining was detected in the cortex or primary xylem (**Figure 5A**). In the middle of the stem strong GUS staining was observed only in the cortex between strands of phloem fiber cells (**Figure 5B**). The stem bottom sections showed a strong staining in the cortex and also in the phloem, especially at the position of the primary rays (**Figure 5C**). Detailed analysis in the mature xylem showed *ARR5::GUS* activity in the ray cells adjacent to vessels (**Figure 6A**). *ARR5::GUS* activity was also detected in the cambial zone (**Figure 6C**).

### *ARR5::GUS* Activity Reports Changes in the Tissue- and Cell-type Specific Pattern of Cytokinin Activity during Dormancy Compared with the Growth Phase

**FIGURE 7:**
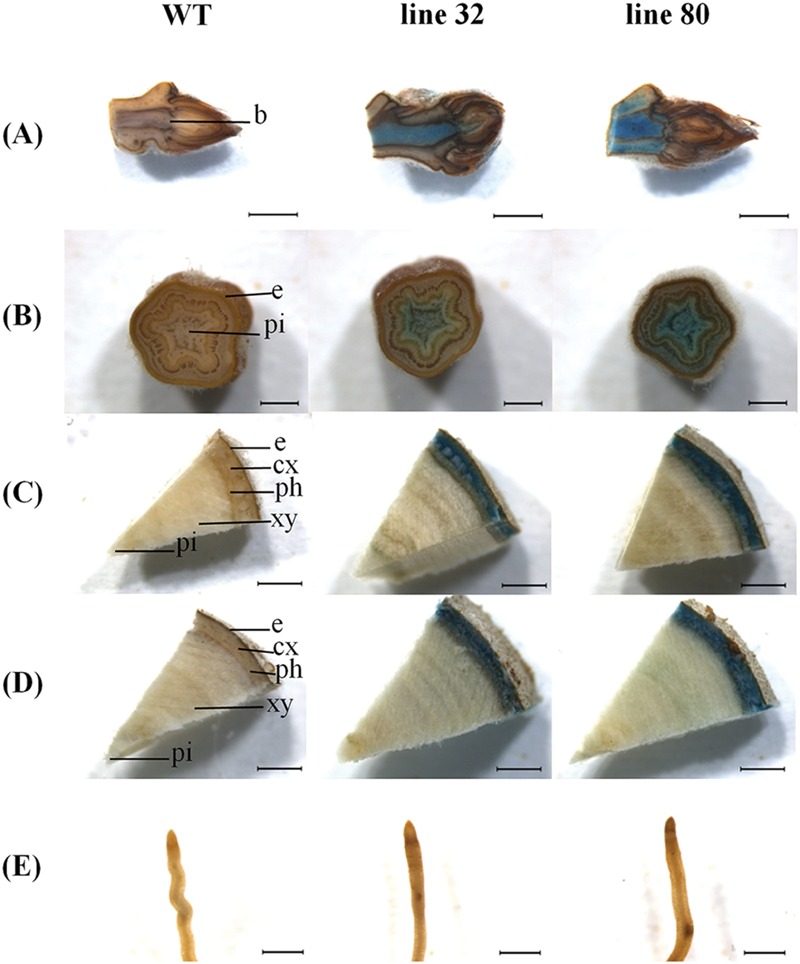
***ARR5::GUS* activity observed in different tissues of *Populus × canescens* reporter lines and WT in winter.** Representative pictures of *n* = 2 replicates per line are shown. Rows **(A–E)** represent apical bud, stem sections from top, middle, and bottom positions, and the root tips, respectively. In the pictures, b, apical bud base; e, epidermis; cx, cortex; ph, phloem; xy, xylem; and pi, pith. Scale bar = 2 mm for apical bud, stem middle, and stem bottom. Scale bar = 1 mm for stem top and root tip.

During winter dormancy the apical buds showed no *ARR5::GUS* activity in the leaf primordia, but a very strong signal in the ground tissue below the bud base (**Figure 7A**). *ARR5::GUS* activity below the bud base extended into a larger area of the ground tissue of the stem than that observed during the growing season.

Below the apex, at the stem top *ARR5::GUS* activity was detected in the pith, however, with weaker intensity than in summer (**Figure 7B**). This signal disappeared in the stem middle and bottom (**Figures 7C,D**). The stem middle and bottom cross sections showed a strong staining in the bark region (**Figures 7C,D**). In the root tips *ARR5::GUS* activity was absent in winter (**Figure 7E**).

**FIGURE 8:**
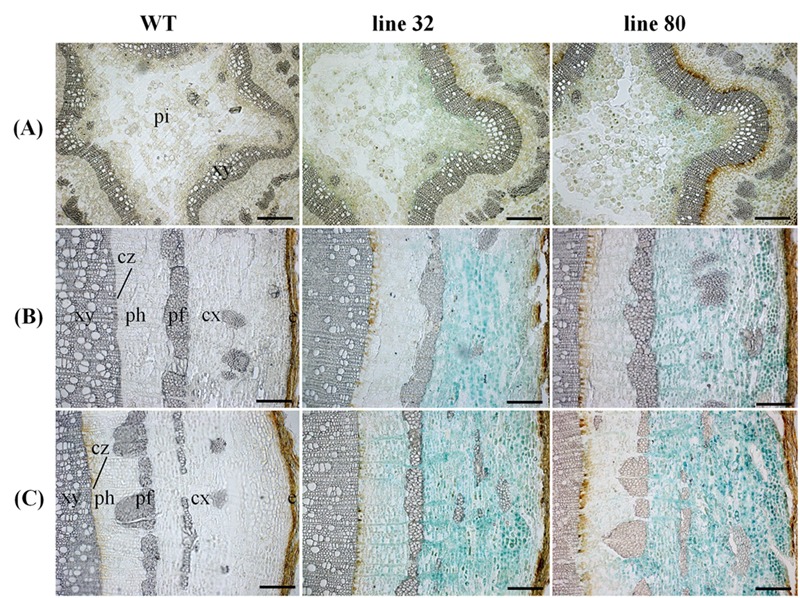
**Cellular localization of *ARR5::GUS* activity in different stem sections of *Populus × canescens* reporter lines and the WT in the growth phase.** Representative pictures of *n* = 2 replicates per transgenic line. Rows **(A–C)** represent stem top, stem middle, and stem bottom sections, respectively. Here e, epidermis; cx, cortex; pf, phloem fibers; ph, phloem; cz, cambial zone; xy, xylem; and pi, pith. Scale bar = 200 μm.

Cellular localization of *ARR5::GUS* activity was also monitored along the dormant stem (**Figure 8**). The stem top section, which was collected at the same position below the apex as in summer, showed a fully developed circular ring of secondary xylem, indicating that secondary growth had already started in this zone (**Figure 8A**). The reason is that after bud set in fall, elongation growth stops and the undifferentiated ground tissues continue to develop for some time. In winter, *ARR5::GUS* activity was present mainly in the outer pith region, the perimedullary zone (**Figure 8A**), whereas in the stem top sections from the growth phase most of the *ARR5::GUS* activity was localized in the center of the pith. In the stem middle, anatomy and the pattern of *ARR5::GUS* activity were similar to that at the stem bottom (**Figures 8B,C**). The *ARR5::GUS* activity extended across the whole cortex region of the bark and therefore, was stronger than in summer in this tissue (**Figures 8B,C**). Similar as in summer, the staining was pronounced in cell files that were connected with xylem rays (**Figures 8B,C**). *ARR5::GUS* activity was also localized in the cambial zone at the stem bottom during dormancy with a stronger signal than that detected in summer (**Figure 6D**).

Similar as in summer, the ray cells adjacent to vessels showed *ARR5::GUS* activity (**Figure 6B**), but the stain was less pronounced than in summer (**Figure 6A**). The *ARR5::GUS* activity in distinct locations of the xylem rays was only present at the stem bottom.

### Tissue Specific Expression Pattern of PtRR Type-A Genes

**FIGURE 9:**
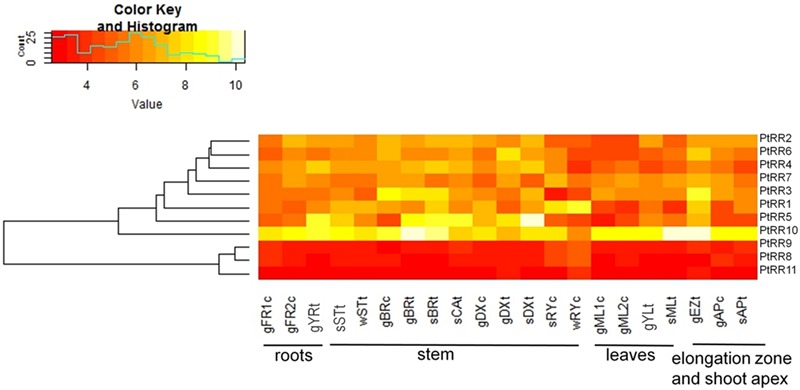
**Tissue-specific expression pattern of poplar genes belonging to the two component response regulator (*PtRR*) type-A gene family of cytokinin signaling pathway.** The following abbreviations were used: FR, fine roots; YR, young roots; ST, stem bottom; BR, bark; CA, cambium; DX, developing xylem; RY, ray cells; ML, mature leaves; YL, young leaves; EZ, elongation zone; AP, shoot apex. The prefixes g, growing plants in controlled conditions; s, summer; and w, winter. The suffix c, tissues from *P. × canescens* and t, tissues from *P. trichocarpa*. The numbers 1 and 2 represent two different experiments. *PtRR9* has no assigned ARR name in *Arabidopsis* (see **Table 1**)

The expression of genes belonging to the type-A RR family in poplar was analyzed in different tissues employing microarray data (**Figure 9**). Each of the 11 genes identified in poplar (Ramírez-Carvajal et al., 2008, cf. **Table 1**) had a probe set on the microarrays and therefore could be included here. There was no clustering of the *PtRR* transcriptional pattern according to tissues (not shown), but all tissues showed an expression of all *PtRR* type-A genes (**Figure 9**). The *PtRR* transcriptional pattern clearly clustered the genes in two categories, one comprising genes with low expression *(PtRR8, 9*, and *11*) and the other with genes that showed variable expression across the tissues and season (*PtRR1, 2, 3, 4, 5, 6, 7*, and *10*). *PtRR10*, the ortholog of *ARR5*, was expressed in all tissues under study, especially in the phloem and elongation zone in the growth phase (**Figure 9**), thus supporting consistency between *PtRR10* expression and our reporter lines. In the fine roots from the growth phase, mainly *PtRR10* was expressed while in the young roots *PtRR5* was also expressed. *PtRR5, 3*, and *1* showed strong expression in the phloem during the growth phase. In the cambium tissues during summer, *PtRR10* and *5* were mainly expressed. In developing xylem, during growth phase, *PtRR10, 5*, and *6* showed strong expression. *PtRR5* showed a strong expression in the developing xylem in summer followed by *PtRR10* and *4*. In the summer rays, *PtRR10* and *1* showed strong expression. *PtRR7* was also expressed in summer ray cells. But in winter rays, only *PtRR1* showed strong expression. In the elongation zone of the stem, during the growth phase, *PtRR10, 5, 3, 6, 1*, and *5* were strongly expressed. In the shoot apex only *PtRR10* was strongly expressed.

## Discussion

### The *ARR5::GUS* Construct Is Functional in Poplar

Many of the biological and developmental phenomena shared by herbaceous and woody plants are regulated by the same molecular mechanism. Besides having the same cytokinin signal transduction components, the type-*ARR* gene family found in *Arabidopsis* and poplar is well conserved in these two plant species (Immanen et al., 2013) with the highest similarity between *PtRR10* and *ARR5* (Ramírez-Carvajal et al., 2008; Immanen et al., 2013). Here, we show that the *ARR5::GUS* reporter construct was functional in poplar because it was inducible by the cytokinin analogs, thidiazuron and BAP and not by adenine, an inactive cytokinin analog. Thus, *ARR5::GUS* transformed poplar lines record the distribution of active cytokinins selectively. However, quantification of the signal is not possible because of the unknown turnover of GUS and the produced indigo dye.

Normal growth of the transgenic poplars indicated that there was no significant non-target effect of the biotechnological modification on plant performance. However, in one of the three reporter lines, *ARR5::GUS* construct was apparently silenced during long-term growth under ambient conditions. Silencing is not uncommon in transgenic plants and can have a number of different reasons (Stam et al., 1997; Fagard and Vaucheret, 2000). The synthetic promoter construct *TCS::GFP* for monitoring cytokinin in *Arabidopsis* was also subjected to silencing (Zürcher et al., 2013). Here, the two active reporter lines showed similar patterns of the *ARR5::GUS* activity in those tissues that also showed *PtRR10* expression, thus, supporting that they confidently recorded cytokinin activity.

### The Localization of *ARR5::GUS* Identifies Novel Cytokinin-Active Cell Types in Poplar

In the growing phase, the main tissues with strong *ARR5::GUS* activity included the apical bud base, the root tips, pith in stem elongation zone, and bark in the stem middle and bottom. The localization of active cytokinins in the apical bud base and in root tips shows strong similarity to that observed in *ARR5::GUS* expressing *Arabidopsis* seedlings (D’Agostino et al., 2000). In *Arabidopsis* seedlings, the primary and lateral root tips showed strong *ARR5::GUS* activity in the root cap region as well as in the cell division region and elongation zone with a gradual decrease toward the direction of the shoot apex (D’Agostino et al., 2000). This staining pattern was also evident in the growing season in poplar root tips in our study. Aloni et al. (2004) reported that the *ARR5::GUS* signal in *Arabidopsis* roots was produced in the statocytes.

In the stem top, the observation of *ARR5::GUS* activity in the pith was unexpected, but similarly Teichmann et al. (2008) also had found auxin activity in this tissue. This finding suggests that the pith may have an important function for the hormone supply in the young stem, where the vascular system is not yet fully differentiated. Active cytokinins were also detected in the cambial zone of poplar, in agreement with studies showing that cytokinins are important regulators of cambial activity in growing poplars (Matsumoto-Kitano et al., 2008; Nieminen et al., 2008).

The presence of active cytokinins in the xylem ray cells, which was detected here, has not been reported so far, but was underpinned by high expression of poplar *PtRR10, PtRR1*, and *PtRR7* in this cell type. A noteworthy finding was that the *ARR5::GUS* activity in the ray cells was seen only in parts associated with vessels. The biological significance of high cytokinin activity close to the vessels is unknown. However, root-derived cytokinin that are transported with the xylem sap through the vessel, are likely to be supplied by this route to the rays.

The *ARR5::GUS* staining pattern observed in the bark cortical cells, primary rays and in the cambium support previous studies reporting that cytokinins are necessary for determining vascular cell identities (Mähönen et al., 2000; Hutchison et al., 2006; Yokoyama et al., 2007; Argyros et al., 2008) and stimulate growth (Werner et al., 2003). The positional pattern of active cytokinin found here agrees with strong expression of the type-*ARR* genes *PtRR3, PtRR5*, and *PtRR10*) in the phloem of greenhouse grown *P. trichocarpa* (Ramírez-Carvajal et al., 2008). Furthermore, we found strong expression of *PtRR10, PtRR5, PtRR3*, and *PtRR1* in bark tissues. In the cambium *PtRR10* and *PtRR5* were highly expressed indicating responsiveness to cytokinins.

The reporter lines generally show expected localization pattern, but an exception was also noted. Although *PtRR10* was expressed in mature leaves, no *ARR5::GUS* activity was found in these tissues. One possibility is that *PtRR10* transcription is regulated by further signals to which *ARR5* is not responsive or the *ARR5::GUS* reporter construct is insensitive to cytokinins in fully expanded leaves in summer. Similar cases have been reported for poplar auxin reporter lines transformed with *GH3::GUS* (Teichmann et al., 2008) and *DR5::GUS* (Chen et al., 2013), where no activity of these constructs was noted in the cambium, a tissue in which an auxin maximum is expected. However, in our study *ARR5::GUS* activity was detected in the leaf primordia, where cytokinin levels determine leaf size (Holst et al., 2011)

A comparison of *ARR5::GUS* activity along the stem with that of auxin reporter lines (*GH3::GUS, DR5::GUS*, Teichmann et al., 2008; Chen et al., 2013) shows overlap of the hormone activities in the elongation zone, but contrasting intensities in the bark. In *ARR5::GUS* poplars, the staining in bark was stronger toward the stem base, whereas that of auxin reporter lines decreased toward the base. These observations indicate that the phytohormone reporter lines also truly reflect the hormone gradients installed by the opposite apical production sites in roots for cytokinins and in the stem for auxin and the inverse transport pattern of these phytohormones along the stem (Jones and Ljung, 2011).

### Cytokinin Activity Is Subject to Seasonal Fluctuations in Distinct Tissues

Seasonal fluctuation of cytokinin activity was most notable in the root tips, the major site of cytokinin synthesis (Aloni et al., 2004, 2005). The absence of *ARR5::GUS* activity in the root tips during dormancy together with a strong presence of *ARR5::GUS* activity in the apical bud base, in the pith, and in the bark suggests that the active cytokinins in these shoot tissues may be shoot-derived rather than root-derived. Cytokinins from different production sites have been distinguished by their chemical composition. Root-derived cytokinins are mainly of the *trans*-zeatin (tZ) type (Aloni et al., 2005), whereas phloem-transported isopentenyladenine (iP) type cytokinins are considered to be shoot-derived (Bishopp et al., 2011). In winter, the concentration of tZ is low in the xylem sap of willows, probably because of their decreased root production (Alvim et al., 1976), which corresponds to the lacking *ARR5::GUS* signal in our study. At the start of dormancy very high levels of the iP type are present in the phloem sap of 14 different tree species (Weiler and Ziegler, 1981). The studies with excised twigs of *Populus × robusta* and rootless almond shoots also confirm the presence of cytokinin after chilling (Hewett and Wareing, 1973; Van Staden and Dimalla, 1981). Increased cytokinin levels of the bark and in buds before the bud burst were further reported in artificially chilled, excised apple shoots (Cook et al., 2001). All these studies suggested that shoot derived cytokinins play a role in dormancy and the following bud burst in spring. The source of these cytokinins could be *de novo* biosynthesis or conversion of storage forms to their active forms (Skene, 1972; Kannangara and Booth, 1974; Van Staden and Brown, 1978; Van Staden, 1979; Van Staden and Dimalla, 1981). Collectively, these studies show that cytokinins are present in the dormant phase and together with our results on *ARR5::GUS* activity, it is clear that they are active in distinct cell types such as cortical and ray cells in the bark, pith, and ray cells next to vessels and in the shoot apex. In the cambial zone, a strong staining was also detected during dormancy. This observation may suggest a role of cytokinins in cambial cell maintenance in winter.

## Conclusion

Employing an *ARR5::GUS* reporter, we monitored seasonal differences and similarities of cytokinin activity at the tissue and cellular level in poplar. Since cytokinins increase the sensitivity of the cambium to the auxin signal, they are important regulators of wood quantity and quality (Aloni, 1991, 2001). Therefore, the reporter lines can be used to investigate the involvement of cytokinins in mediating growth constraints and growth-promoting treatments for vascular development and cell type identities in the future. Thereby, these poplars may become an important tool to enhance our understanding of woody biomass production.

## Author Contributions

SP conducted field and laboratory experiments, analyzed data, and wrote the manuscript. HW supervised experiments, analyzed data, and commented on manuscript. DJ analyzed bioinformatic data and commented on the manuscript. TT constructed the vectors, characterized transformants, and commented on the manuscript. RH transformed plants, tested the transformants, and commented on the manuscript. AP designed the experiments, analyzed data, and wrote the manuscript. All authors contributed to the final version of the manuscript.

## Conflict of Interest Statement

The authors declare that the research was conducted in the absence of any commercial or financial relationships that could be construed as a potential conflict of interest.

## Footnotes

1 http://genome.jgi-psf.org/Poptr1_1/Poptr1_1.home.html

2 http://phytozome.jgi.doe.gov/pz/portal.html

3 www.arabidopsis.org
